# Validity of the Capacity to Work Index: Development of an Instrument to Measure Work Capacity in Relation to Depression and Anxiety in the General Working Population

**DOI:** 10.1007/s10926-023-10150-2

**Published:** 2023-11-08

**Authors:** Gunnel Hensing, Cornelia van Diepen, Maria Boström, Monica Bertilsson

**Affiliations:** 1 Social Medicine, School of Public Health and Community Medicine, Institute of Medicine, The Sahlgrenska Academy at the University of Gothenburg, Gothenburg, Sweden; 2 Centre for Person-centred Care, The Sahlgrenska Academy, University of Gothenburg, Gothenburg, Sweden; 3 Erasmus School of Health Policy and Management, Erasmus University Rotterdam, Rotterdam, The Netherlands; 4 Social Medicine, School of Public Health and Community Medicine, PO Box 453, SE-405 30 Göteborg, Sweden

**Keywords:** Work capacity, Work functioning, Common mental disorders, Psychometric testing, Health care professionals

## Abstract

**Purpose:**

To develop an index to assess capacity to work in relation to common mental disorders (CMDs) in the general working population and field test its psychometric properties.

**Methods:**

Content analysis of three qualitative studies on people (*n* = 49) with their own experiences of working with CMD guided the items selected for the index. Face and content validity and test-retest reliability were performed. The index was field tested in two versions with 26 and 17 items, respectively, among health care professionals regarding internal reliability, component structure and concurrent validity.

**Results:**

The final version of the Capacity to Work Index (C2WI; 17 items) was normally distributed in the field test with high internal reliability (Cronbach’s alpha, 0.84). Missing responses were randomly distributed and nonspecific. Principal component analysis showed one clear component with negatively framed items. Concurrent validity showed high correlation with the WHO-5 Well-Being Scale (Pearson’s *r*, 0.68), but lower correlation for the general health question (*r*, − 0.44), one item of the Work Ability Index (*r*, − 0.33), and the Stress of Conscience constructs (*r*, 0.44).

**Conclusion:**

The C2WI showed promising psychometric qualities. Low and negative correlation with the item from Work Ability Index suggests that the C2WI measures additional dimensions, but further testing in larger and more diverse samples is required.

## Introduction

Common mental disorders (CMDs) are major public health problems in the general working population with consequences for work performance, decreased productivity, and safety risks at work [1, 2]. Most people with CMD continue to work supported by medication, psychotherapy or self-supporting techniques; others have reduced capacity to work leading to sickness absence [3]. The latter situation puts pressure on universal and other social security schemes by increasing costs and presents challenges in the management of back to work rehabilitation. Medical and other treatments for CMDs reduces the patient’s symptoms but in many cases, symptom reduction is not sufficient to regain capacity to work [4]. When followed longitudinally, people with depression and anxiety in remission still showed decreased work performance [5, 6]. A general assumption is that symptoms of CMD lead to reduced capacity to work. However, an initial reduction of capacity to work may lead to CMD symptoms, or symptoms and reduced capacity to work may trigger each other with a downward negative spiral [7]. More knowledge on this process is needed for future development of preventive measures. Research based in working populations, representing a wide spectrum of capacities and symptoms, is most appropriate to achieve this compared to for example sick-listed or clinical samples with reduced capacity to work and/or symptoms to an extent that led to care seeking. No specific instrument to assess capacity to work in relation to CMDs in a general working population is available. Most instruments have been developed for clinical use for individuals with CMD seeking care [8–11].

Capacity to work consists of several dimensions related to each other in a dynamic and complex way. Commonly used models such as the PEOM (Person, Environment, Occupation Model) or the WAI (Work Ability Index) include person-related factors, the work environment and work tasks [12, 13]. Each of these dimensions include several factors. A qualitative interview study with Swedish physicians described the assessment as doing a jigsaw puzzle without a master model [14]. A literature review of qualitative studies regarding physicians’ assessment of work capacity in people with CMD in the clinical situation also identified difficulties: individual variations in symptoms, how these affect the patient at work, and the physician’s trust in the patient’s own story of reduced capacity to work [15]. People with their own experience of CMD also find it difficult to explain why they cannot work. A phenomenological study identified lost familiarity with the ordinary work performance, putting up a working facade, and time-consuming new practices to manage work as essential in the capacity to work while depressed and anxious [16]. Another study explored work instability and identified a process of working in dissonance when the ordinary fluency at work was disturbed [17]. The process included experiences of work as performed out of rhythm, in discomfort, disconnected and with an experience of working in a no-man’s land. Other studies have described difficulties concentrating on work, thinking clearly, handling the workload, and interacting with other people [18, 19]. A review of how CMDs affect health care professional’s work found difficulties in keeping up, coping with emotions, and lack of energy as common [1]. These studies show the complexity and dynamic nature of capacity to work with CMDs.

In epidemiologic studies of occupational health, the concept of capacity to work in relation specifically to CMDs has received less attention than generic concepts [20, 21]. A possible reason is the lack of specific and relevant instruments for use in research on working populations. The generic Work Ability Index (WAI) is a commonly used instrument for assessment of work ability in general working populations. It is comprehensive, reflects physiological and psychosocial aspects of work ability, and has good psychometric properties [22]. It was originally developed to assess work ability in an ageing perspective, and to explore the role of work demands in work ability [12]. However, as noted by Ilmarinen [12] the concept of work ability has developed over the years in a more holistic and versatile direction, and that there are several research questions that need to be addressed. He mentions health and functional capacities, and the fact that severity of a disease not always aligns with reduced work capacity. And perhaps, even more challenging the question why not all persons with good health are able to work. This latter question is highly relevant in relation to common mental disorders where studies have found low association between reduction of symptoms and return to work [5, 6]. Apart from the need for complementary perspectives and research on the work ability construct, some methodological drawbacks have been noted. They include its two-dimensional construct; some questions relate to “a subjective” or perceived component of work ability and others to “an objective” health-related component [22–24]. The objective component includes diagnosis and sickness absence. The subjective component, apart from self-assessment of work ability, includes questions about mental well-being and thoughts about the future [22, 23]. It has been argued that this conceptual distinction needs to be resolved [23]. The Work Role Functioning Questionnaire [8] was developed to assess workers’ ability in relation to job demands in relation to health status. The instrument measures different types of demands and can be used in various occupational groups and in relation to different clinical conditions. The instrument is not specifically developed for common mental disorders, but a section covers mental and social demands. The Mini ICF-APP [11] is an instrument developed for clinical assessment of work capacity and mental disorders integrating assessments of symptoms and capacities. Self-reports from patients are used together with information from case reports and clinical observations. The Lam Employment Absence and Productivity Scale (LEAPS) was developed as a tool for clinical use to assess and monitor work functioning in the specific group of patients with major depressive disorders [10]. The Work Instability Scale for People with Common Mental Disorders (WIS-CMD) was developed to measure work instability described as early signs of reduced function for use at occupational health services, work places or primary care [9]. The existing instruments, WAI, WRFQ, mini-ICF-APP, LEAPS, and WIS-CMD, measure to different degrees generic assessments of work capacity in the general population and/or in clinical populations. LEAPS is mainly intended as a clinical instrument for a specific diagnostic group (MDD), and the mini-ICF-APP also has a clinical perspective (including case reports and clinical observations). WAI is mainly a generic assessment of work capacity and not developed for any diagnostic group. WRFQ has also a generic profile covering work schedule, output, physical, mental/social, and flexibility demands at work. The assessments can be combined with various diagnostic groups. Finally, the WIS-CMD focuses on work instability defined as early signs of reduced functioning which might imply that it is less adapted for assessment in a mixed working population with different levels of work capacity.

Existing instruments cover important aspects of work capacity, but none has specifically focused on common mental disorders in a general working population perspective. There are important differences between the composition of the study populations in clinical and epidemiological studies [20, 21]. In clinical studies, be it in occupational health services, primary health care or in hospitals, the participants have been identified as having symptoms or a diagnosis of CMD. In cases of return to work for sick-listed patients, the capacity to work is assessed as reduced by a physician. Thus, the variation in work performance in a clinical study population can be expected to be lower than in a general working population, which includes people with no CMD symptoms and full capacity to work as well as those with both subthreshold and significant symptoms and reduced capacity to work. Another reason for developing a new instrument was to focus more directly on *why* people cannot work as complementary to the *demands* and *symptoms* a person experiences. With more knowledge on *why* we hope that preventive measures can be developed with more precision. The instrument in this study was developed to provide a capacity to work instrument in relation to CMD in the general working population to gain more knowledge on the co-development of CMD and capacity to work. It can be used to follow-up preventive interventions at work given the role of psychosocial factors at work for the development of CMD.

There is a need for better understanding of how capacity to work and symptoms of CMD interact in a general working population, and to explore why some people can continue to work with CMD while others with similar severity of CMD are sick listed. Currently, there is no instrument to assess capacity to work in this respect. The aim of this study was to develop an index for assessment of capacity to work in relation to CMD in the general working population and to field test its psychometric properties in terms of content and concurrent validity and test-retest reliability.

## Methods

The study is part of the research programme “New Ways—Mental health at work”. The first part of the development and field-testing of the C2WI investigated whether the content and format of the items was acceptable. The second part field tested the index in a general working population for validity and general applicability. The first part of this study was approved by the Regional Ethical Review Board (no. 783-16). The Swedish Ethical Review Authority approved the second part of this study (no. 2019-01287).

### Part 1

#### Development of the Questions in the C2WI

We developed the items for the C2WI from three qualitative studies that explored work and CMD (Table 1) [16, 17, 25].

**Table 1:** Characteristics of the three Swedish qualitative studies used to identify meaning units from which items for the capacity to work index were developed [16, 17, 25]

Aim	I	To explore experiences of capacity to work in persons working while depressed and anxious in order to identify the essence of the phenomenon capacity to work
	II	To explore and describe health care professionals’ experience-based understanding of capacity to work in individuals with depression and/or anxiety disorders
	III	To explore experiences of work instability in workers with common mental disorders
Study design	I	Phenomenological
	II	Explorative qualitative, content analysis
	III	Grounded theory
Type of interview	I	Focus groups (*n* = 4)
	II	Focus groups (*n* = 4)
	III	Individual in-depth interviews (*n* = 27)
Participants (*N* = 65)	I	Persons working at least part-time with illness experiences of common mental disorders or clinically diagnosed (*n* = 17; 12 women and 5 men)
	II	Health care professionals from occupational, psychiatric, and primary health care (*n* = 21; 15 women and 6 men)
	III	Workers with common mental disorders (*n* = 27; 19 women and 8 men)
Meaning units identified	I	26
	II	15
	III	10

The first, a phenomenological study, explored the essence of capacity to work while depressed and anxious [16]. This study identified nine constituents that served as a framework for development of the items; all constituents should be represented in the index by at least one item. The second study, a content analysis, explored health care personnel’s understanding of capacity to work in patients with depression and anxiety [25]. The third study, a grounded theory study, explored the process of work instability in people with depression and anxiety [17]. As a first step, one of the co-authors (Ma.B.) did an independent manifest content analysis of the three studies to identify meaning units, that is text sections or sentences that contains information reflecting aspects of capacity to work [26].

Similarities and differences of all meaning units identified (*n* = 69) were discussed continuously by Ma.B., M.B., and G.H. until consensus was reached. Overlapping meaning units (*n* = 18) that reflected the same content but in different wordings were excluded. Unique units (*n* = 51) were included for the index (Table 1). Transformation of the meaning units into items in the index resulted in 48 preliminary items. For quality control, Ma.B. compared the preliminary items with the units from the analyses and identified another meaning unit that was transformed into a preliminary item. Thus, the first version of C2WI consisted of 49 items.

A decision was made to use the time frame “during the last week” based on a combined consideration of recall bias and a theoretical assumption of the dynamic nature of capacity to work in relation to CMD [16, 17]. The items had five response alternatives: “not at all”, “to a low degree”, “to a moderate degree”, “to a high degree”, and ”I don’t know/not relevant”.

The last response category was added because not all items corresponded to the varying work environments and work tasks (e.g., learning new tasks and meeting new people). Moreover, some items might be difficult to interpret (e.g., put on a facade) and require an “I don’t know” response category. Four response categories were a pragmatic choice adapted to customary practice as recommended by Statistics Sweden (www.scb.se) to manage a middle answer alternative.

#### Face Validity of the First Version of the Index

Thirty-three clinicians and researchers from the authors’ professional networks, and with different experiences of or knowledge in CMD, were invited to help reduce the number of items. Of these, 22 participated, read the 49 items, and gave feedback on their relevance, intelligibility, content, and the priority of the items in the first version of the C2WI. An example of an item considered less relevant and overlapping with other items was *I have felt clumsy, which has made it difficult for me to do my work tasks.* A couple of other items did also reflect bodily sensations (dizziness, nausea) and their influence on work capacity. We decided to group these into a more overarching item. The final item representing this is number 15: *I have felt weak, sore or tense in my body, which has hindered me in my work*. Other items considered less relevant reflected level of work pace such as *I have been working at an increased pace.* Items reflecting work pace were grouped into: *I have been able to keep the pace of work required in my work.* Based on this feedback, and our reasoning, we reassessed the 49 items and reduced them to 26 items. The chosen response alternatives were considered relevant and retained.

#### Content Validity of the Second Version of the Index

The user-friendliness of the items was tested in a random sample of 50 employees in two departments of a large Swedish municipality. Managers of the departments were initially contacted by e-mail to set up a telephone meeting. They were informed about the study, approved participation and provided work e-mail addresses. The employees were contacted by the researchers through an e-mail. They were informed that the study was independent of the employer and participation was voluntary after informed consent. Informed consent was given by answering and sending back the questionnaire. The sample included clerical officers/assistants, civil servants, librarians, and managers. The questionnaire consisted of the 26 items in the second version of the C2WI, demographic questions and three questions on user-friendliness. The first question dealt with how easy it was to understand the items, with four response alternatives: “very easy to understand”, “fairly easy to understand”, “fairly difficult to understand”, and “very difficult to understand”. The next two questions had open response alternatives and addressed difficulties with understanding the questions, and if the response alternatives suited the items. The first question on user-friendliness covered the whole questionnaire. Specific items could be addressed in the two open-ended questions.

Twenty-seven employees (54% of the sample) responded. Overall, the index showed good user-friendliness with understandable questions and suitable response alternatives. Seven items had a robust uneven distribution of responses. These items were reviewed, which resulted in deletion of four items and rephrasing of the other three. Examples of items that were deleted were *I have had difficulty taking in information (written or verbal) and I have felt that I have performed my work in a mechanical way.* One item was considered too general and was deleted on that basis. This process resulted in a third version of the C2WI with 21 items.

#### Test-Retest Reliability

A new random sample (*n* = 70) was drawn from the employees in the same municipality, but from a different department, for assessment of test-retest reliability. The procedure was the same as above. The manager provided work e-mail addresses. The employees were informed that the study was independent of the employer and that participations was voluntary after informed consent by replying to the questionnaire. They were also informed that the test included two questionnaires with an interval of approximately 14 days. This time interval was considered suitable for assessing work capacity; not too short to remember the earlier answers and not too long for possible changes at work [27].

As in the previous test, the first test consisted of C2WI and demographic questions. The demographic questions were not repeated in the retest, which consisted only of the C2WI and one new question. The new question concerned whether something had happened at work between the test and retest that could influence the subsequent results. Nineteen employees (27.1%) completed both questionnaires. This number was too low for statistically valid tests. The results were used instead for manual assessment of further exclusion of items in combination with expert opinion of the researchers. This resulted in the exclusion of another five items, reducing the index to 16 items; the items were: *I have had difficulty concentrating on my tasks. I have forgotten about things. I have had difficulty getting started with my tasks. I’ve had to double check myself to make sure I’ve done it right. I have experienced anxiety, worry or anxiety which has hindered me in my work.* This version was tested by research fellows and social networks (*n* = 33) to assess timing and get general comments. This led to one item, *I have been able to keep my composure and not wind up*, being split into two to clarify the content (see item 16 and 17 in Table 2). Thus, the final index included 17 items (Table 2). The development of the items was thereby completed and ready for an initial field test of the C2WI [28].

**Table 2:** Distribution of responses to the capacity to work index in relation to common mental disorders in a sample of Swedish health care professionals, 2019–2020

Items	Not at all, *n* (%)	To a low degree, *n* (%)	To a moderate degree, *n* (%)	To a high degree, *n* (%)	Mean	SD’	Not relevant/ I don’t know, *n*^a^	Missing, *n*
1. Disturbing sounds have hindered me in my work	20 (39.2)	13 (25.5)	12 (23.5)	6 (11.8)	2.08	1.06	3	7
2. Thinking has been tough and slow	13 (25.5)	23 (45.1)	12 (23.5)	3 (5.9)	2.10	0.86	1	9
3. I have had a hard time prioritizing my tasks	17 (33.3)	27 (52.9)	5 (9.8)	2 (3.9)	1.84	0.76	1	8
4. I have been able to keep the pace of work required in my work	3 (5.9)	5 (9.8)	8 (15.7)	35 (68.6)	3.47	0.90	1	7
5. I have had difficulty controlling my emotions	24 (47.1)	23 (45.1)	3 (5.9)	1 (2.0)	1.63	0.69	4	2
6. I have been sensitive to criticism from people I have met	25 (49)	18 (35.3)	6 (11.8)	2 (3.9)	1.71	0.83	4	5
7. I have “put on a facade” to be at work	20 (39.2)	16 (31.4)	9 (17.6)	6 (11.8)	2.02	1.03	4	4
8. I have continued to work even though I have experienced mental or physical problems	23 (45.1)	9 (17.6)	10 (19.6)	9 (17.6)	2.10	1.17	5	2
9. In order to be able to work, I have had to scale things back outside of work	20 (39.2)	11 (21.6)	15 (29.4)	5 (9.8)	2.10	1.04	2	6
10. I have received energy and job satisfaction from my duties	4 (7.8)	5 (9.8)	28 (54.9)	14 (27.5)	3.02	0.84	3	3
11. I have had a hard time learning new tasks	29 (56.9)	17 (33.3)	4 (7.8)	1 (2.0)	1.55	0.73	3	5
12. I have felt like a stranger at work	41 (80.4)	7 (13.7)	1 (2.0)	2 (3.9)	1.29	0.70	3	4
13. I’ve been “like in a bubble” which has hindered me in my work	33 (64.7)	14 (27.5)	3 (5.9)	1 (2.0)	1.45	0.70	3	3
14. I have avoided occasions when many (people) meet because I have not been able to participate	21 (41.2)	15 (29.4)	12 (23.5)	3 (5.9)	1.94	0.95	4	3
15. I have felt weak, sore or tense in my body, which has hindered me in my work	23 (45.1)	16 (31.4)	9 (17.6)	3 (5.9)	1.84	0.93	2	5
16. I have been able to keep my composure	4 (7.8)	7 (13.7)	9 (17.6)	31 (60.8)	3.31	0.99	2	4
17. I have been easily wound up	15 (29.4)	14 (27.5)	14 (27.5)	8 (15.7)	2.29	1.06	3	4

‘*SD* standard deviation

^a^Data from the 11 excluded participants that did not answer all statements of the C2WQ or had at least one statement with “not relevant/ I don’t know”

### Part 2

#### Study Design of the Initial Field Test

The 17-item C2WI was included in a survey to health care professionals in six departments of three Swedish hospitals. The survey assessed work-related health after the introduction of a model of Person-Centred Care [29]. The sample was not chosen to represent specific experiences in relation to CMD. Instead, the choice was made based on the opportunity to test the instrument in a sample representing one sector of the general working population in an ongoing research project.

#### Context and Study Participants

The study involved self-reported data collected at baseline and follow-up at 6 and 12 months. Recruitment in hospital departments took place in autumn 2018 and included two cardiology departments, a geriatrics and rehabilitation department, pulmonary department, orthopaedics department, and surgery. The staff numbers varied from 14 to 57.

#### Data Collection

**Fig. 1:**
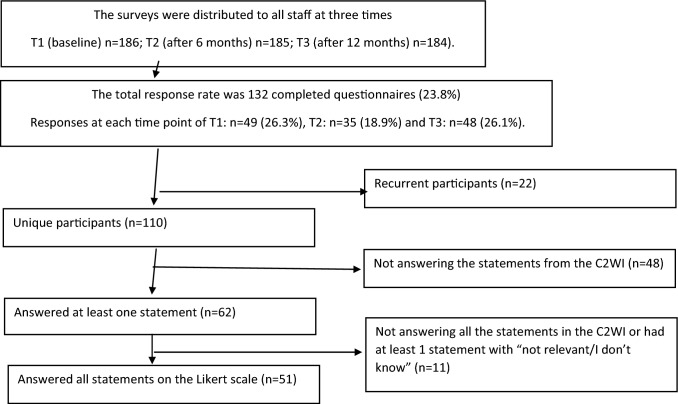
Flowchart of the data collection and response rates in the initial field study of the capacity to work index (C2WI), 2019–2020

The survey included several instruments such as the WHO-5 Wellbeing scale, the Stress of Conscience Questionnaire, items from the Work Ability Index and the C2WI. The survey also included questions, not analysed in this study, such as measuring demand and control at work and experiences of working with Person-Centered Care [29]. It took approximately 20 min to fill in the survey. The surveys were distributed to all staff at three times. The flowchart of eligible participants is provided in Fig. 1. Data were collected using a standardized online self-report questionnaire in Swedish. A link to the questionnaire was sent to the work e-mail addresses of the health care staff in the participating departments. All the participants had a unique online ID, could respond at any time, and withdraw from the study at any time. The online survey programme collated the self-reported data and unidentified the entries for data processing. Due to a low response rate, the responses from the three collection points were combined to increase the number of entries for analysis. As participants could respond on all three occasions, the second and third questionnaires had the additional question: “Have you answered this questionnaire before?” The entries with a positive response to this question were eliminated in the present study to create a sample of unique participants. Furthermore, the participants needed to have answered all the items in the C2WI (see flowchart in Fig. 1 and distribution of responses in Table 2). Thus, the final sample for the initial field-testing consisted of 51 unique individuals. These individuals had no missing data in any of the instruments included in this study. The same sample was used for all tests of the C2WI in the initial field test reported in this study.

#### Instruments Used in the Field Test of Concurrent Validity

Concurrent validity refers to testing the C2WI against comparable measures to establish that the index correlates with other measures but also provides proof of the need for this new index. Four constructs were selected for the tests of concurrent validity: (1) The WHO-5 Well-Being Scale based on a summation score of the five statements about well-being [30]; (2) the self-rated general health question: How would you rate your overall health? with a Likert scale from 1 to 10 [31]; (3) one item from the WAI: How would you rate your current workability compared with the lifetime best? with a Likert scale from 1 to 10 [32]; and (4) the Stress of Conscience questionnaire consisting of eight items evaluating the frequency of a selected stressful situation using a scale ranging from 1 (never) to 10 (every day) and the perceived degree of troubled conscience generated by the situation using a scale ranging from 0 (no, not at all) to 10 (yes, it gives me a very troubled conscience) [33]. These measures were expected to have some degree of overlap with the C2WI but provide different angles that could help determine the validity of the C2WI within this field. As mentioned above the study was performed as part of a survey on work-related health after the introduction of a model of Person-Centred Care. The choice of comparable instruments was limited to the measures used in the survey.

### Statistical Analyses

As part of the psychometric evaluation, descriptive analysis was performed to go through the distribution and response patterns. The internal consistency was estimated by Cronbach’s alpha. Principal component analysis (PCA) was done to test the assumption of uni-dimensionality. Pearson’s correlation coefficient analyses were done to evaluate concurrent validity. The analyses were performed using IBM SPSS Statistics (IBM Corporation, 2018) and a *p* value < 0.05 was considered significant. The C2WI items needed to be recoded to create an overall index and for the psychometric analyses. Items 4, 10, and 16 were positively framed and were reversed. The answer option “I don’t know/not relevant” was coded as value missing for the creation of the index.

## Results

The field tests were based on a sample of 51 health professionals consisting mainly of female nurses. Table 2 gives an overview of how the responses were distributed. Most respondents selected the more positive ends of the response scale. As an example, forty-one of the study sample of 51 answered “not at all” to item 12: “I have felt like a stranger at work”. Between 1 and 9 participants selected the worst response alternatives for the different items. For item 8 “I have continued to work even though I have experienced mental or physical problems”, the response alternative “to a high degree” was selected by nine participants. Finally, the last two column presents the distribution of missing over the items among excluded respondents. The addition of the “I don’t know/not relevant” option showed the anticipated result and participants could report that the item did not apply to their situation. However, this option was not applied consistently; a random pattern was seen. Different participants chose this option in different instances emphasizing that this option was an individual choice and not consistent within the workplace.

The descriptive statistics for the C2WI are presented in Table 3.

**Table 3:** Descriptive statistics of the capacity to work index in relation to common mental disorders (C2WI)

Descriptive statistics	
No. of respondents	51
Range of item scores	17–68
Minimum score	18
Maximum score	49
Mean score	30.5
Standard deviation	8.0
Skewness	0.28
Kurtosis	− 0.79
Cronbach’s alpha	0.84

The C2WI was normally distributed. It was reasonably symmetrical (slightly right skewed) with a slightly flatter (platykurtic) distribution. The internal reliability measured with Cronbach’s alpha was 0.84. The scores when the individual items were removed did not alter the internal reliability to an extent that would suggest removing that item from the C2WI.

### Principal Component Analysis

PCA was performed to estimate the extent to which the structure of the multi-item C2WI adequately reflected the hypothesized uni-dimensionality of the construct, and to evaluate if a reduction in the number of items was possible without losing too much information. The PCA is presented in Table 4 with a four-component solution and eigenvalues. The PCA showed a clear single component solution in which the negatively framed items of the C2WI were excluded. Items 1, 11, and 12 had a value below 0.5, which is below acceptability within one dimension. However, no decisions to reduce the number of items were taken in this initial field study given the low number of participants and homogenous professional group.

**Table 4:** Principal component analysis of the capacity to work index

Component matrix	Components (eigenvalue per component)			
	1 (5.86)	2 (1.50)	3 (1.44)	4 (1.27)
8. I have continued to work even though I have experienced mental or physical problems	0.860			
9. In order to be able to work, I have had to scale things back outside of work	0.793			
7. I have “put on a facade” to be at work	0.753			0.356
14. I have avoided occasions when many (people) meet because I have not been able to participate	0.739		0.342	
15. I have felt weak, sore or tense in my body, which has hindered me in my work	0.712		0.419	
17. I have been easily wound up	0.678			
3. I have had a hard time prioritizing my tasks	0.653		− 0.392	
13. I’ve been “like in a bubble” which has hindered me in my work	0.639	− 0.502		
2. Thinking has been tough and slow	0.626	0.332		
6. I have been sensitive to criticism from people I have met	0.576			
5. I have had difficulty controlling my emotions.	0.573		0.460	
12. I have felt like a stranger at work	0.341	− 0.646	0.318	
10*. I have received energy and job satisfaction from my duties*		0.355	0.565	− 0.494
1. Disturbing sounds have hindered me in my work	0.341	0.434		0.525
11. I have had a hard time learning new tasks	0.436			− 0.313
4*. I have been able to keep the pace of work required in my work*		− 0.342		0.393
16*. I have been able to keep my composure*				0.407

Items in italic font are reversed

Correlation, >0.3; eigenvalue, >1.2

### Concurrent Validity

The concurrent validity was tested by correlating the C2WI with four other constructs. The C2WI was significantly and positively related to the WHO-5 Well-Being Scale (*r* = 0.68; *p* < 0.001). Correlation with the single general health correlation was negative but *r* = − 0.44 (*p* = 0.002) was less than 0.5. C2WI and WAI had a negative correlation, and *r* = − 0.33 (*p* = 0.02) indicating low correlation. Correlation with the Stress of Conscience construct was positive (*r* = 0.44; *p* = 0.003) but again *r* was < 0.5, indicating low correlation. In summary, the results of the correlation analysis showed promising concurrent validity. Expressed differently, we suggest that the construct of C2WI measures a new dimension of capacity to work, and that it reflects aspects of mental health shown by correlation with WHO-5 Well-Being Scale.

## Discussion

The aim of this study was to develop a questionnaire specifically for assessment of capacity to work in relation to CMD and adapted for use in general working population samples. The aim was also to do an initial field test of psychometric properties. As discussed in the introduction, instruments of high quality exist, covering relevant content and with good psychometrics. Some of the instruments are generic (WAI, WRFQ) and not specifically developed for CMD while others are developed for clinical use assessing patients (LEAPS, mini-ICF-APP) [8, 10–12]. The instruments cover demands at work and/or symptoms. Knowing that different demands at work contribute to CMD and sickness absence with CMD are important both in preventive work and in vocational rehabilitation [34]. However, knowing even more in detail *why* the capacity to work is reduced would improve the efforts to create CMD-friendly workplaces; such workplaces would probably benefit all employees. The C2WI is an attempt to provide an instrument that can be used to follow a group of employees to see if specific items are predictive for future sickness absence, if predictive items are similar over occupations and different types of work environment. Comparisons should be done between groups of employees with similar levels of symptoms from CMD to come closer understanding why some persons can work with CMD while others cannot [5, 6]. The index was developed from a content analysis of findings in three qualitative studies of individuals with their own experience of working with CMDs. This was done to promote content validity, e.g., that the index measures experiences of relevance in relation to working with CMD. The index was adapted after tests of validity and reliability in two samples representing the intended future target groups (the general working population).

### Promising Results for the Validity and Field Tests

Our overall assessment of this first validity and field test is that the results are promising. In particular, the uni-dimensionality of the construct and the results of the concurrent validity tests were important. The uni-dimensionality was not complete because a couple of items had *r* < 0.5, suggesting that these were representing another dimension. There are a couple of ways to handle this. One would be to delete the items and stay with a 14-item solution, which would be tempting from a response rate perspective. Fewer items might increase the participation rate. However, from a theoretical point of view, we found it relevant to keep the three items (1, 11, and 12). These items mirror experiences that have been found in interview studies to be important among those with experiences of CMDs and work. Experiences of being a stranger or feeling alienated at work have been found in different studies [16, 17, 35]. A Dutch study in a clinical population found associations between self-perceived sensitivity to noise and increased psychological distress, decreased general health and higher prevalence of prescriptions for antidepressants and benzodiazepines [36]. Cognitive difficulties seem to be a major symptom in both depression and exhaustion disorders [37, 38]. So, these items capture aspects of capacity to work in relation to CMDs that are less prevalent, possibly due to a late appearance in the process from performing well at work to a reduction in capacity to work. Thus, no items were deleted, and the 17-item version of the C2WI was kept.

Promising concurrent validity was also an essential finding. The C2WI correlated significantly with the WHO-5 Well-Being Scale, which supports that C2WI captured aspects related to the continuum of mental health to ill health. In a future study with a larger sample, an analysis of item differences in correlation strength would be important.

The correlation between the WAI and the C2WI was low and negative (*r = −* 0.33). One objective of the C2WI research was to complement existing measurements with an instrument specifically developed to capture aspects related to CMDs. Thus, the negative association found in this study is promising and might reflect aspects not captured by the WAI. However, it has also been argued that when the WAI is limited to one question, it might not capture the full range of work ability [23]. El Fassi et al. [32] found that poor work ability measured with WAI was associated with physical job tasks, but mental work tasks had a favourable impact on work ability. Future studies need to explore this more closely, but a possible reduced range of WAI in relation to content might explain the low and negative correlation between C2WI and WAI. If this finding can be repeated in future studies, it supports the need for a new measure for work capacity in CMDs.

### The Complexity of Assessing Capacity to Work in Relation to CMDs

Research that has explored how physicians and other health care professions manage the assessment of work capacity for CMDs concluded it is a complex process [15]. A focus group study with physicians in Sweden concluded that it was like putting together a puzzle without a model and that the final result was a highly individualized assessment for that specific patient [14]. Other studies have highlighted the dynamic nature of CMD and related capacity to work [16, 17, 25]. The symptoms may vary from one day to another, and the capacity to work may also vary and is dependent on the specific tasks and social context during a workday. Many studies are based on clinical assessments with care-seeking patients. To assess capacity to work in a general working population is even more challenging due to the larger spectrum ranging from mental health (the majority) to mental ill health (the few). A construct must be able to catch at least some aspects of both ends. Thus, it is difficult to develop a construct that captures the full range of aspects related to the capacity to work. However, there is a great need to further develop the research field on capacity to work and CMDs. Given the high prevalence in working populations, an important future research question is how symptoms of CMDs and capacity to work are interrelated. The assumption is often that CMD comes first and leads to reduced capacity to work, but it might be the other way around or a parallel negative, downward spiral [7]. To really understand this, we need studies assessing capacity to work in general working populations. Such studies are also needed to follow universal preventive interventions at workplaces targeting not only CMDs but also the capacity to work. C2WI can facilitate such studies.

### Strengths and Limitations

A main strength is that three qualitative interview studies was used to identify relevant content for the C2WI construct. Focus group interviews are a common approach for item-banking. Because these are seldom published, it cannot be guaranteed they are analysed as comprehensively as published qualitative studies. The literature has suggested that the initial item-banking should be at least twice the final scale; in our study, we initially identified almost three times the final number of items [39]. The first steps in the development of the C2WI also had several check points with clinical professionals and researchers to ensure content and face validity. A limitation was that we could not include people with experience in the early phases of the development. Despite contacts with several primary health care centres, no patients applied for participation in the initial phase of the study.

Another strength is that the early tests with general working populations contributed to a reduction of items and improved understandability. Unfortunately, we did not reach enough numbers to do a proper test-retest analysis. Future studies are needed to assess reliability, even though that is challenging due to the dynamic nature of capacity to work and CMD.

For the C2WI, we chose the last week as time reference. This was done with the target group of a general working population in mind. For people with fairly good work capacity and mental health, it is probably more difficult to remember fluctuations in capacity to work over time. This time frame seemed to work well for the field test. Earlier studies with clinical populations have time frames from 1 week to 1 month [20].

The index has four response alternatives and a fifth possibility (“not relevant/I do not know”). Research on how to adapt questionnaires from a respondent perspective and promote participation rates support the inclusion of a “not relevant/I do not know” response alternative. It is frustrating for the respondent to not be able to reply correctly. In a general working population, the variation in work situations is large and some items might not reflect reality. For example, those who work alone cannot refer to social encounters, and thus item 14 is not relevant.

In the analysis, we included only those who replied to one of the four response alternatives. This is a conservative approach recommended by Statistics Sweden to avoid bias. A consideration was made to include participants who missed 1 or 2 items, but most of the 11 participants who did not respond to all items had missed more than this. Thus, we chose a conservative approach in this psychometric testing and only included the responses to the complete Likert scale.

In future studies with the C2WI, we recommend that the response options “not relevant” and “I do not know” are separated into two different options, thus having six response alternatives. The main reason for this is to take the respondents’ perspective and meet the need to tick a suitable answer [40]. We recommend that these responses are omitted from the analysis of capacity to work according to C2WI. However, they could be useful in future analyses of the possibility to reduce the number of items. So far, we suggest that it is also relevant to retain items with low variation to capture individuals who might be most at risk for work instability or future sickness absence.

A limitation was that the sample for the field-testing was smaller than anticipated, which reduced the statistical strength of the results. The sample was also homogeneous with mainly female nurses. However, health care personnel at hospitals have the advantage of representing a variety of occupational tasks (e.g., technical, emotional, social, and cognitive). Thus, capacity to work can be affected in different ways. A professional sample with more monotonous work tasks would probably have less variation in their capacity to work experiences. Overall, the findings from the field test were congruent and clear and we have no reason to believe that the associations would be completely different in another sample.

Future studies with larger and less homogeneous samples are needed to further test the C2WI. Of special interest would be to compare C2WQ with other instruments such as the full WAI and the WRFQ. For future analyses, this study provides support for formulating hypotheses regarding for example concurrent validity. This study presented the development of the index and first field tests on internal validity and the general performance of the index. Future studies are needed to test the external validity and to develop an understanding of how C2WI is associated with work instability, reduced work performance and sickness absence. Such knowledge could also better inform us on possible cut-off levels for minor or major reduction of work capacity in relation to CMDs.

## Conclusion

The aim of this study was to develop an index for assessment of capacity to work in relation to CMDs in the general working population and perform initial field-testing of its psychometric properties. The C2WI was based on qualitative studies on individuals with their own experience of working with CMDs to ensure content validity. The index was adapted after tests of validity and reliability in two samples representing the intended future target groups (the general working population). The initial field test of the index in a sample of hospital professionals showed satisfactory validity with normal distribution and high internal reliability, random distribution of missing responses, one clear component and promising concurrent validity. The correlation between C2WI and the WAI was negative, suggesting that the C2WI measures additional dimensions. The positive correlation with the WHO-5 Well-Being Scale points to similarity in reflecting mental aspects. The C2WI needs further testing in larger and more diverse samples, and in other contexts, norms, and welfare systems.

## Data Availability

The data used to develop the C2WI are available in published papers by Bertilsson et al. [16], Bertilsson et al. 2015 [25], and Danielsson et al. [17]. Datasets generated during and/or analysed during the field study (part two in this article) are available from the corresponding author on reasonable request.
